# The Association Between Personality Traits and Depressive Symptoms in U.S. Chinese Older Adults

**DOI:** 10.1093/geroni/igaa057.1264

**Published:** 2020-12-16

**Authors:** Maggie Li, XinQi Dong, Dexia Kong

**Affiliations:** 1 Rutgers University, Chicago, Illinois, United States; 2 Rutgers University, New Brunswick, New Jersey, United States

## Abstract

Depressive symptoms are prevalent in the aging population and can negatively impact the health and well-being of older adults. Personality traits may interact with depressive symptoms, but there is currently limited knowledge regarding this relationship in minority aging research. This study aims to explore the associations between two personality traits, neuroticism and conscientiousness, and depressive symptoms in 3,157 U.S. Chinese older adults. Data were obtained from the Population Study of Chinese Elderly in Chicago (PINE) collected between 2011 and 2013. Neuroticism and conscientiousness were measured by the NEO Five-factor Inventory. Depressive symptoms were measured by the nine-item Patient Health Questionnaire (PHQ-9). 45.3% of the participants reported at least one depressive symptom. Controlling for potential confounders, logistic regression analyses showed that both traits were significantly associated with depressive symptoms. One unit increase in neuroticism was associated with 19% increased odds of having any depressive symptoms (odds ratio [OR]=1.19, 95% confidence interval [CI]=1.17-1.22). One unit increase in conscientiousness was associated with 5% decreased odds of having any depressive symptoms (OR=0.95, 95% [CI]=0.94-0.96). Results validate the significant association between both traits and depressive symptoms among U.S. Chinese older adults, adding to the psychological and cultural profiles of those who have experienced mental distress. More in-depth examination using culturally-tailored measurements for personality traits is encouraged in minority aging studies. The NEO inventory was developed from Western populations and hence might not adequately represent personality traits valued by non-Western cultures.

